# Data supporting the functional role of Eleven-nineteen Lysine-rich Leukemia 3 (ELL3) in B cell lymphoma cell line cells

**DOI:** 10.1016/j.dib.2017.09.042

**Published:** 2017-09-22

**Authors:** Lou-Ella M.M. Alexander, January Watters, Jessica A. Reusch, Michelle Maurin, Brook S. Nepon-Sixt, Katerina Vrzalikova, Mark G. Alexandrow, Paul G. Murray, Kenneth L. Wright

**Affiliations:** a Cancer Biology Ph.D. Program, University of South Florida, Tampa, FL 33612, United States; b Department of Immunology, H. Lee Moffitt Cancer Center and Research Institute, Tampa FL33612, United States; c Department of Molecular Oncology, H. Lee Moffitt Cancer Center and Research Institute, Tampa, FL33612, United States; d Institute of Cancer and Genomic Sciences, University of Birmingham, Birmingham, United Kingdom

**Keywords:** ELL, Eleven-nineteen Lysine-rich Leukemia, EBV, Epstein Barr Virus, BL, Burkitt's Lymphoma, ELL3, Transcription elongation, B-cell Lymphoma, Cell division, EBV

## Abstract

The data presented here are related to the research article entitled “Selective expression of the transcription elongation factor ELL3 in B cells prior to ELL2 drives proliferation and survival” (Alexander et al., 2017) [1]. The cited research article characterizes Eleven-nineteen Lysine-rich Leukemia 3 (ELL3) expression in the B cell compartment and functional dependence in B lymphoma cell lines. This data report describes the mRNA expression pattern in a panel of cell lines representing the B cell compartment, supplementing the protein expression data presented in the associated research report. In addition, a reanalysis is presented of publicly available mRNA expression data from primary murine B cells to reveal dynamic regulation of the ELL family members post LPS stimulation (Barwick et al., 2016) [2]. The effect of ELL3 depletion on cell morphology, latent Epstein Barr Virus (EBV) lytic replication and differentiation markers in a Burkitt's lymphoma (BL) cell line cells are presented.

**Specifications Table**

**Table:** 

Subject area	Immunology and Molecular Biology
More specific subject area	Transcriptional elongation
Type of data	Figures and Images
How data was acquired	– quantitative PCR (qPCR) (Bio-Rad CFX96 Real-Time PCR Detection System and CFX96 Software) – Reanalyzed publically available RNA-Seq experiment GSE70294 – Time lapse imaging (Evos Auto FL Cell Imaging System and Image Studio Software) – Western blot (SDS-Page gel electrophoresis and wet transfer; Bio-Rad equipment and Bio-Rad clarity chemiluminescent detection)
Data format	Analyzed
Experimental factors	– RNA was extracted from untransduced cell line model cells and expression assessed – RNA-Seq experiment was done on LPS treated primary murine B cells that were cell sorted by divisions and CD138 levels. – Namalwa BL cell line transduced with control and two independent mCherry-tagged ELL3 specific shRNA's for five consecutive days – Protein and mRNA was extracted from Namalwa BL cell line transduced with control and two independent mCherry-tagged ELL3 specific shRNA's for five consecutive days and expression assessed.
Experimental features	– Quantitative mRNA detection of ELL, ELL2 and ELL3 in B cell compartment cell line models – Expression levels of ELL, ELL2 and ELL3 were extracted from the data set GSE70294 of RNA-Seq performed on each Cell Titer Violet and CD138-positive, populations following LPS stimulus of murine primary B cells. – shRNA transduced Namalwa cells were imaged at day 6 post transduction every 5 min for 24 h. – Western blot analysis of PRDM1 levels and detection of PRDM1, EBV lytic replication genes (BZLF1, BMRF and BLLF1), B cell factors (BCL6, PAX5, MYC) and plasma cell factor (membrane bound and secreted IgM) mRNA levels
Data source location	H. Lee Moffitt Cancer Center and Research Institute, Tampa, FL, USA
Data accessibility	Data is within this article

**Value of the data**

• This data describes an expression pattern of ELL family members that is replicated in both human and murine B cell compartment
• The data shows the role of ELL3 in the morphology of B cells and reveals disruption of cell division
• The data reveals the impact of ELL3 depletion on B cell differentiation markers and latent EBV gene expression.

## 1 Data

**Fig. 1:**
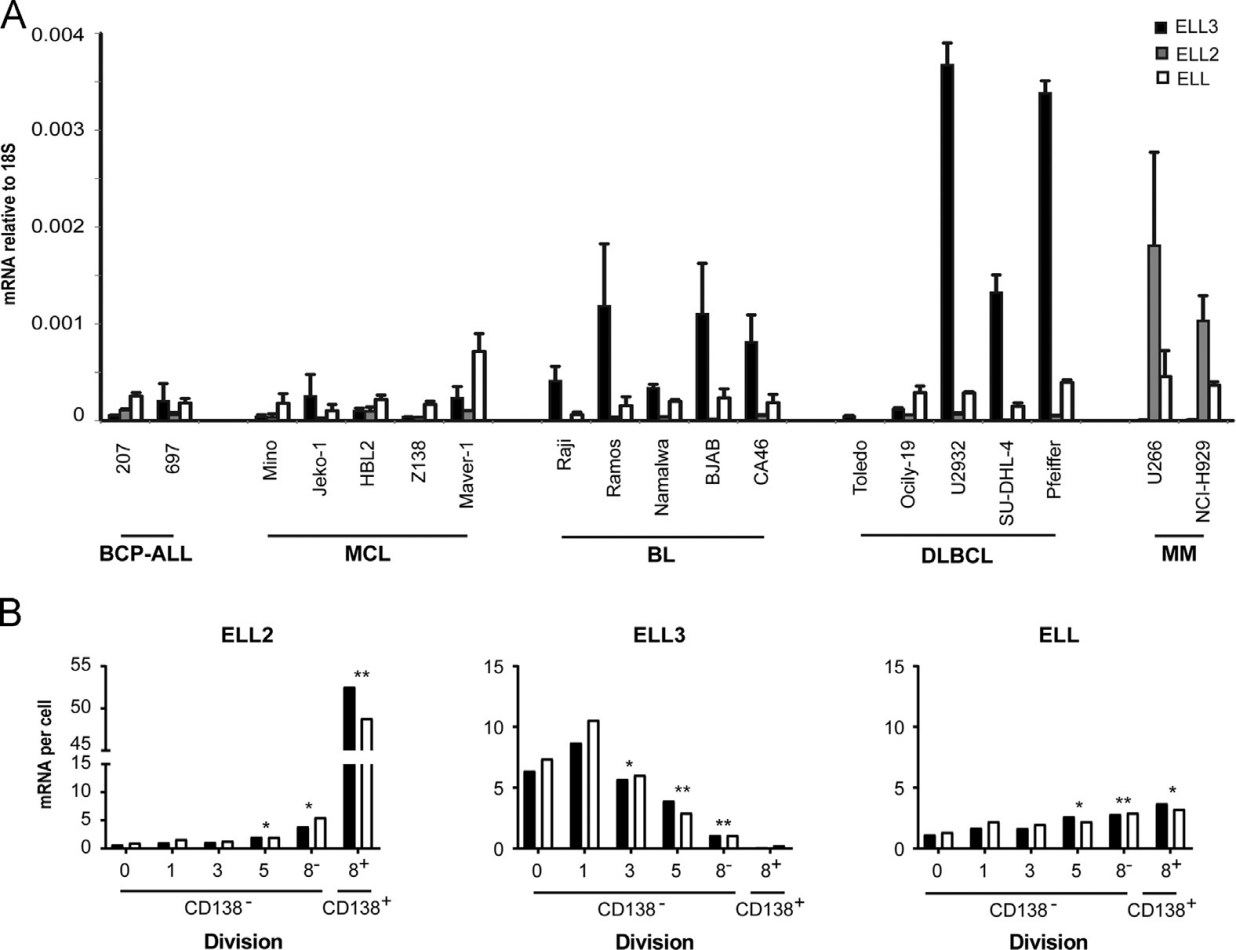
ELL family member transcript levels in murine primary B cells and human B cell lymphoma cell lines. A. Relative mRNA expression profile of ELL family members across human B cell lines. The cell line name and lymphoma subtype are indicated on the x-axis. Data represents the average of 3 independent experiments; errors bars represent SD. B. Expression of ELL family members mRNA in murine B cells during proliferation and differentiation in response to LPS stimulation in vivo. Data was extracted from GSE70294 [2] and presented as mRNA copies per cell. The x-axis represents the number of cell divisions. CD138 positivity is indicated by the (+) and represents the fully differentiated plasma cells. Data represents one experiment with biological duplicates. *p<0.05, **p<0.01 (*two-tailed t-test*).

**Fig. 2:**
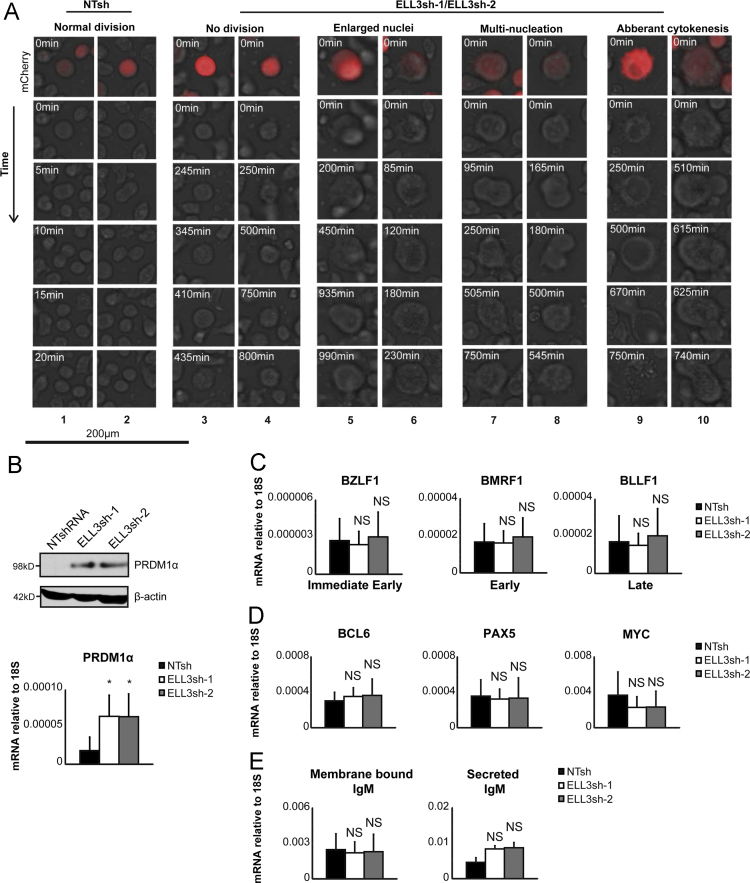
Effects of ELL3 depletion on EBV lytic replication, B cell differentiation and morphology. ELL3 expression was depleted in Namalwa cells by transduction with either NTsh, ELL3sh-1, or ELL3sh-2 shRNA expression constructs co-expressing mCherry. A. At day 6 post transduction, cells were subjected to time lapse imaging. Images were taken every 5 min. over 24 h. Data depicts representative images of the control and ELL3-depleted cells. The mCherry fluorescence signal (red) was used to identify shRNA transduced cells. Subsequent images are from the same cell but only imaged with phase to facilitate observation of morphological changes. Time of acquisition is indicated in each image. **B.** Protein and relative PRDM1α mRNA levels detected by RT-qPCR and immunoblot at 5 days post transduction. C. The relative mRNA quantitation of the EBV genes; BZLF1, BMRF1 and BLLF1. D. The relative mRNA quantitation of B cell factors; BCL6, PAX5 and MYC. **E.** The relative mRNA quantitation of membrane bound and secreted IgM. Data in panels B through E is presented as the average of 5 independent experiments; errors bars represent SD. *p<0.05; NS is not significant (*two-tailed t-test*).

The mRNA levels of ELL, ELL2 and ELL3 in a B cell lymphoma cell line panel is depicted in Fig. 1A. Fig. 1B depicts the average mRNA per murine primary B cell following LPS stimulus and cell sorting based on cell division and plasma cell marker CD138 based on data from GSE70294 [2]. The effect of ELL3 depletion is shown in Fig. 2; including observations of cell morphological changes, PRDM1 mRNA expression, EBV lytic replication factors expression, B cell factors BCL6, PAX5, MYC, and immunoglobulin isoforms.

## 2 Experimental design, materials and methods

### 2.1 Cell culture

Cell lines and cell culture details are as described previously [1].

### 2.2 Lentiviral shRNA knockdown

The shRNA vectors MISSION® TRC2 pLKO.5-puro ELL3shRNA (ELL3sh-1, TRCN0000289149; and ELL3sh-2, TRCN0000296220), and MISSSION® TRC2 pLKO.5-puro Non-Mammalian control shRNA (NTsh; SHC202; Sigma Aldrich, St. Louis, MO) were purchased. The existing puromycin resistance gene in these vectors was replaced with the mCherry tag from the pLVmCherry vector (Addgene, Cambridge, MA). We produced lentiviral particles with the jetPRIME transfection reagent (Polyplus transfection, Illkirch, France) in HEK-293T using 3rd generation lentiviral packaging mixture (Applied Biological Materials Inc., Richmond, Canada). Cells were transduced at 5×10^7^ cells/ml for 2 h at 1500*g*, room temperature (RT) in the presence of 0.6 µg/ml polybrene (Merck Millipore, Billerica, MA). Functional assessments were done five days after transduction.

### 2.3 Immunoblotting

Immunoblotting procedure was as described previously [3]. Primary antibodies include: β-actin (1:12,000 dilution) (AC-15, Sigma Aldrich, St. Louis, MO) and PRDM1 (C14A4). Horse radish peroxidase conjugated secondary antibodies were purchased from GE Healthcare Life Sciences (Pittsburgh, PA).

### 2.4 Quantitative mRNA analysis

RNA was extracted using the E.Z.N.A. Total RNA Kit I (Omega Bio-Tek, Norcross, GA) and reverse transcribed into cDNA with the qScript cDNA synthesis Kit (Quanta Biosciences Inc., Gaithersburg, MD). 3 µl of one to eleven diluted cDNA was analyzed in duplicate using primers specific to PRDM1α, EBV lytic replication genes (BZLF1, BMRF1 and BLLF1), and B cell and plasma cell factors (BCL6, PAX5, MYC, membrane-bound IgM, secreted IgM) at primer specific annealing temperatures. mRNA expression was analyzed using the ΔΔC_t_ method, with 18 S as a normalization gene [4]. Primer sequences are described in Supplemental Table I and were designed to span exon-exon junctions and to amplify a single PCR product [3, [5], [6], [7], [8], [9]]. The annealing temperatures were experimentally determined using a temperature gradient and high efficiency was validated by PCR of cDNA serial dilutions.

### 2.5 Microscopy

For time-lapse imaging, cells were plated on a 6-well flat bottom plate at 2×10^5^ cells/ml, placed in Evos Onstage Incubator set at 37 °C and 20%O_2_ and imaged every 5 min for 24 h on Evos Auto FL Cell Imaging System (Thermo Fisher Scientific Inc., Waltham, MA). All images were taken at 20×magnification using the RFP filter and phase.
