# Supplementary material for: Data supporting the functional role of Eleven-nineteen Lysine-rich Leukemia 3 (ELL3) in B cell lymphoma cell line cells
Source: Data Brief. 2017 Sep 22;15:222–7. doi: 10.1016/j.dib.2017.09.042 (PMC5633249; doi:10.1016/j.dib.2017.09.042)
Supplement: Supplementary file 2 — Supplementary material [file mmc2.docx]

**Supplemental Table I: Primer sequences**

| **Primer** | **Sequence (5’🡪3’)** | **Reference** |
| --- | --- | --- |
| 18S | FWD: CGGCTACCACATCCAAGGAAGG  REV: CCCGCTCCCAAGATCCAACTAC | [[3](#_ENREF_3)] |
| ELL | FWD: CTGGGCAAGGTTCAGTT  REV: CACTCGCCAAGTTGATGG | _ |
| ELL2 | FWD: AGAGTCTCCTGAGTGGTTCGTC  REV: AAAGGCCAAGATGTCCAAGA | _ |
| ELL3 | FWD: ACCTGACTGAAGATGCCAGA  REV: ACTGTCCTTGGTTGCTTGC | _ |
| PRDM1α | FWD: TACATACCAAAGGGCACACG  REV: TGAAGCTCCCCTCTGGAATA | [[5](#_ENREF_5)] |
| BZLF1 | FWD: CGCCTCCTGTTGAAGCAGAT  REV:AAATTTAAGAGATCCTCGTGTAAAACATC | [[6](#_ENREF_6)] |
| BMRF1 | FWD: CAACACCGCACTGGAGAG  REV:GCCTGCTTCACTTTCTTGG | [[7](#_ENREF_7)] |
| BLLF1 | FWD: ACTCATTATCACACGAACGG  REV: ATCCAGTTGTATTCAAGGTAGG | [[8](#_ENREF_8)] |
| MYC | FWD: GGAACGAGCTAAAACGGAGCT  REV: GGCCTTTTCATTGTTTTCCAACT | + |
| BCL6 | FWD: CACCATCCCTTTTTGAAGTG  REV: AACGCGGTAATGCAGTTTAG | + |
| PAX5 | FWD: TGGAGGATCCAAACCAAAGG  REV: GGCAAACATGGTGGGATTTT | [[9](#_ENREF_9)] |
| Membrane bound IgM | FWD: GTGTCCGAAGAGGAATGGAA  REV:GTTCTCAAAGCCCTCCTCGT | _ |
| Secreted IgM | FWD: GTGTCCGAAGAGGAATGGAA  REV: ATGACCAGGGACACGTTGTA | _ |

-*Primers were designed by the authors, + Primers purchased from realtimeprimers.com.*
